# Diurnal variations of brown fat thermogenesis and fat oxidation in humans

**DOI:** 10.1038/s41366-021-00927-x

**Published:** 2021-08-02

**Authors:** Mami Matsushita, Shinsuke Nirengi, Masanobu Hibi, Hitoshi Wakabayashi, Sang-il Lee, Masayuki Domichi, Naoki Sakane, Masayuki Saito

**Affiliations:** 1 Department of Nutrition, Tenshi College, Sapporo, Japan; 2 Division of Preventive Medicine, Clinical Research Institute, National Hospital Organization Kyoto Medical Center, Kyoto, Japan; 3 Biological Science Research Laboratories, Kao Corporation, Tokyo, Japan; 4 Laboratory of Environmental Ergonomics, Faculty of Engineering, Hokkaido University, Sapporo, Japan; 5 Department of Biomedical Sciences, School of Veterinary Medicine, Hokkaido University, Sapporo, Japan

**Keywords:** Obesity, Fat metabolism

## Abstract

**Background/objectives:**

Disturbed circadian rhythm is associated with an increased risk of obesity and metabolic disorders. Brown adipose tissue (BAT) is a site of nonshivering thermogenesis (NST) and plays a role in regulating whole-body energy expenditure (EE), substrate metabolism, and body fatness. In this study, we examined diurnal variations of NST in healthy humans by focusing on their relation to BAT activity.

**Methods:**

Forty-four healthy men underwent ^18^F-fluoro-2-deoxy-D-glucose positron emission tomography and were divided into Low-BAT and High-BAT groups. In STUDY 1, EE, diet-induced thermogenesis (DIT), and fat oxidation (FO) were measured using a whole-room indirect calorimeter at 27 °C. In STUDY 2, EE, FO, and skin temperature in the region close to BAT depots (Tscv) and in the control region (Tc) were measured at 27 °C and after 90 min cold exposure at 19 °C in the morning and in the evening.

**Results:**

In STUDY 1, DIT and FO after breakfast was higher in the High-BAT group than in the Low-BAT group (*P* < 0.05), whereas those after dinner were comparable in the two groups. FO in the High-BAT group was higher after breakfast than after dinner (*P* < 0.01). In STUDY 2, cold-induced increases in EE (CIT), FO, and Tscv relative to Tc in the morning were higher in the High-BAT group than in the Low-BAT group (*P* < 0.05), whereas those after dinner were comparable in the two groups. CIT in the High-BAT group tended to be higher in the morning than in the evening (*P* = 0.056).

**Conclusion:**

BAT-associated NST and FO were evident in the morning, but not in the evening, suggesting that the activity of human BAT is higher in the morning than in the evening, and thus may be involved in the association of an eating habit of breakfast skipping with obesity and related metabolic disorders.

## Introduction

Whole-body energy expenditure (EE) exhibits diurnal variations, depending largely on the effects of physical activity and food intake. There have been reports that the thermic effect of food intake, the so-called diet-induced/postprandial thermogenesis (DIT), is higher in the morning than in the evening and at night [1–6]. This may explain the apparent association of meal timing with obesity and related metabolic disorders: for example, habits of breakfast skipping and night eating may lead to increased body fat accumulation probably because of decreased DIT and daily EE [7–11]. However, the mechanism for the diurnal variations of DIT is poorly understood in humans despite the possible involvement of an endogenous circadian system [12].

Brown adipose tissue (BAT) is the major site of nonshivering thermogenesis (NST) during cold exposure [cold-induced thermogenesis (CIT)] in small rodents [13]. Since the rediscovery of metabolically active BAT using [^18^F]fluorodeoxyglucose positron emission tomography and computed tomography (FDG-PET/CT) in adult humans [14–17], it has been confirmed that human BAT is activated by cold exposure and contributes to the increase of whole-body EE and fatty acid oxidations, and thereby to the regulation of body fat [18–20]. Although cold exposure is undoubtedly the most physiological and effective regimen to activate and recruit BAT, increasing exposure to cold temperatures in our daily life would be difficult and uncomfortable. DIT is another component of NST. We previously reported that DIT is ~50% higher in subjects with metabolically active BAT than in those without it, thus suggesting a significant contribution of BAT to DIT in humans [21]. Actually, the activation of BAT after meal intake is directly confirmed by PET/CT using [^15^O]O_2_, [^15^O]H_2_O and [^18^F]fluoro-thiaheptadecanoic acid radiotracers [22]. This leads us to hypothesize that BAT may be involved in the diurnal variations of DIT. To test this idea, in the present study, we reanalyzed our previous 24 h EE data from subjects with and without metabolically active BAT [21]. In these analyses, we focused on DIT and fat oxidation (FO) after breakfast, lunch, and dinner (STUDY 1). In a separate series of experiments, to examine whether human BAT exhibits diurnal variations in vivo, we examined cold-induced responses of whole-body EE and skin temperature in the supraclavicular region, which are likely surrogates of activated BAT, in the morning and evening (STUDY 2). Here we show diurnal variations of BAT activity in humans, which is involved, at least in part, in higher DIT and fatty acid oxidation in the morning.

## Methods

### Subjects

Twenty-one (STUDY 1) and 23 (STUDY 2) healthy men were recruited through poster advertisements and word of mouth. All participants provided written informed consent before study commencement. The protocol of STUDY 1 was approved by the institutional review boards of the National Institute of Health and Nutrition, Tenshi College, National Center for Global Health and Medicine, and Kao Corporation, Japan. The protocol of STUDY 2 was approved by the institutional review boards of Tenshi College and Kyoto Medical Center, Japan.

### ^18^F-FDG-PET/CT

BAT activity was measured using FDG-PET/CT as reported previously [14]. Briefly, after fasting for 10–12 h, subjects wore light clothes (T-shirt and shorts) and remained in a room wherein the temperature was adjusted to 19 °C for 2 h. Intermittently, a towel-wrapped ice block was placed against the soles of their feet. After 1 h, ^18^F-FDG (1.66–5.18 MBq/kg body weight) was intravenously administered and the subjects remained in the same cold conditions for another hour. One hour after ^18^F-FDG administration, a PET/CT scan was performed at 24 °C using a dedicated PET/CT system (an Aquiduo [Toshiba Medical Systems, Otawara, Japan], Biograph 16 [Siemens Medical Solutions, Knoxville, TN, USA], or Discovery PET/CT 600 [GE Healthcare, Waukesha, WI, USA]). Detectable FDG uptake into the supraclavicular BAT was assessed by visually judging. In parallel, the FDG uptake was semiquantitatively measured as the maximal standardized uptake value (SUV_max_). The SUV_max_ threshold level between the detectable and undetectable was 2.00 [14].

### Anthropometrics and body composition analysis

Body composition was measured by either whole-body dual-energy X-ray absorptiometry (QRD 4500 W, Hologic Inc., Waltham, MA, USA) or by the multifrequency bioelectric impedance method (HBF-361, Omron Healthcare, Kyoto, Japan).

### STUDY 1: Diurnal changes in BAT-associated DIT and fat oxidation

Subjects and methods were previously reported [21]. Briefly, 21 healthy men (20–50 years of age with a body mass index (BMI) of 18.0–24.9 kg/m^2^) were divided into two groups: those with visually undetectable FDG uptake with SUV_max_ of 1.1 ± 0.4 (Low-BAT group, *n* = 8), and those with detectable FDG uptake with SUVmax of 8.5 ± 4.8 (High-BAT group, *n* = 13). No significant difference was found between the two groups regarding age, anthropometric, and blood parameters (Table 1 in ref. [21]).

**Table 1:** Subject characteristics.

	Low BAT	High BAT	*P* value
Number	8	15	
Age (years)	23 ± 3	23 ± 1	0.81
Weight (kg)	64.5 ± 9.0	61.8 ± 7.2	0.44
BMI (kg/m^2^)	22.0 ± 2.9	21.0 ± 2.1	0.36
FFM (kg)	53.7 ± 5.2	51.1 ± 5.3	0.27
Fat mass (kg)	10.8 ± 4.7	10.7 ± 3.0	0.94
SUVmax	0.79 ± 0.47	8.04 ± 4.74	<0.001

Values are mean ± SD.

*BAT* Brown adipose tissue, *BMI* body mass index, *FFM* fat-free mass, *SUVmax* maximal standardized update value.

Twenty-four-hour calorimetric measurements starting from 0000 h were performed with a whole-room indirect calorimeter (Fuji Medical Science Co. Ltd, Chiba, Japan) Briefly, the subjects entered the calorimeter at 1900 h and went to bed at 0000 h and woke up at 0700 h. The time from 0715 to 0900 h was used for rest and a low-intensity activity program. During the remaining time, free activities were allowed to emulate daily living conditions. They ate the same meal in exact the same volume at 0900 h (breakfast), 1400 h (lunch), and 1900 h (dinner), and they were allowed to drink water freely. The meal contains 15 energy percent (E%) protein, 25 E% fat, and 60 E% carbohydrate. Energy intake was individually adjusted from the basal metabolic rate based on subject age, sex, height, and body weight, and multiplied by 1.3 as the limited physical activity level.

The air in the chamber was pumped out at a rate of 100 L/min. Temperature and relative humidity of the incoming fresh air were maintained at 27.0 ± 0.2 °C and 50.0 ± 3.0%, respectively. The air samples were dried using a gas-sampling unit (CPF-8000, Shimadzu Corp., Kyoto, Japan) and analyzed with a mass spectrometer (VG PRIMA δB, Thermo Fisher Scientific, Cheshire, UK). Based on oxygen consumption, carbon dioxide production, and urinary nitrogen excretion, the total EE (TEE), respiratory quotient (RQ), fat oxidation, and carbohydrate oxidation were calculated. DIT was calculated by plotting TEE against the physical activity according to the method of Schulz et al. [23].

### STUDY 2: Diurnal changes in cold-induced EE, fat oxidation, and thermogenesis

Twenty-three healthy men (20–29 years of age and BMI of 19.3–24.1 kg/m^2^), none of them participated in STUDY 1, underwent FDG-PET/CT, and were divided into two groups, those with visually undetectable FDG uptake with SUV_max_ of 0.79 ± 0.47 (Low-BAT group, *n* = 8), and those with detectable FDG uptake with SUVmax of 8.04 ± 4.74 (High-BAT group, *n* = 15). No significant differences between the two groups were found regarding their ages and anthropometric parameters (Table 1).

Whole-body EE and skin temperature at 27 °C and 90 min after cold exposure at 19 °C [24, 25] were measured in the morning (0800–1100 h) and again in the evening (1900–2200 h) 11–13 h after fasting. In brief, the subjects wore light clothing (usually a T-shirt with underwear) and relaxed on a bed at 27 °C. Oxygen consumption and carbon dioxide production were recorded for 20 min with the use of a respiratory gas analyzer connected to a ventilated hood (AR-1, Arco System, Kashiwa, Chiba, Japan). The steady-state value of the last 10 min period was used to calculate the basal EE and RQ. The subjects then underwent infrared thermography with a thermal imaging camera (DE-TC1000T; D-eyes Inc., Osaka, Japan). The skin temperature at the supraclavicular region (Tscv) was measured from each image. The skin temperature at the chest region (Tc) just laterally to the sternum was simultaneously measured as a control. Then, the subjects moved to a cold room maintained at 19 °C, and after 90 min, respiratory gas parameters were recorded for 20 min. Because protein oxidation remains unaltered by mild-cold exposure [26], oxygen consumption and carbon dioxide production were adjusted for those by estimated protein oxidation (21.4% of resting EE at 27 °C) and used for the calculation of fat and carbohydrate oxidation, as described previously [24]. CIT and cold-induced oxidation of fat were calculated from the difference between the values at 27 and 19 °C.

### Statistical analysis

Values are presented as the mean ± standard deviation (SD). Statistical analysis was performed with SPSS (version 26, IBM Japan, Tokyo, Japan). The differences in the participant profiles between the High- and Low-BAT groups were compared with Student’s *t* tests. The values of EE, DIT, RQ, and fat oxidation after meal intake, and those of skin temperature, EE, CIT, and fat oxidation after cold exposure were analyzed using analysis of variance for repeated measures based on a within-subject factor (clock time) and a between-subject factor (BAT). Post hoc multiple comparisons were conducted by the Tukey’s post hoc test. *P* values of <0.05 were considered significant.

## Results

### STUDY 1: Diurnal changes in BAT-associated DIT and fat oxidation

**Fig. 1:**
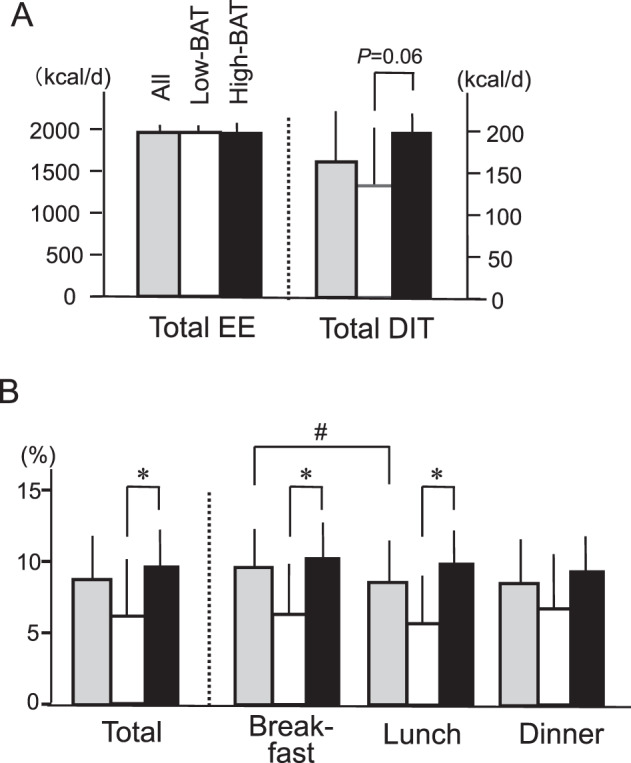
Energy expenditure (EE) and diet-induced thermogenesis (DIT) after breakfast at 0900 h, lunch at 1400 h, and dinner at 1900 h. **A** Total EE per day and total DIT for 15 h after three meals. **B** DIT expressed as % of energy content of the meal. Black columns, High-BAT group; white columns, Low-BAT group; gray columns, all. Values are mean ± SD. *P < 0.05 between the groups, ^#^*P* < 0.05 between the meals.

A part of the results of STUDY 1 has previously been reported [21]. Briefly, the mean EE of all subjects during sleep (SMR) was 1437 ± 107 (kcal/d). This was strongly and positively correlated with the individual fat-free mass (FFM) (*r* = 0.803, *P* < 0.001). The mean DIT (%) for 15 h (0900–2359 h) of all subjects was 8.55 ± 0.75% of energy intake. When comparing of the High-BAT group with the Low-BAT group, there was no significant difference between the groups in TEE, SMR, TEE/FFM, and physical activity levels. DIT expressed as kcal/day tended to be higher in the High-BAT group (Fig. 1A). DIT (%) calculated as the normalized ratio of DIT with the energy intake was higher (*P* < 0.05) in the High-BAT group than in the Low-BAT group (Fig. 1B).

To analyze the impact of BAT and meal timing on the postprandial responses, DIT was calculated during a 5 h period after breakfast, lunch, and dinner, based on the 24 h profiles of EE (Fig. 2 in ref. [21]). DIT in all subjects was significantly higher after breakfast than after lunch, but not after dinner (Fig. 1B). This seems consistent with the previously reported diurnal variations of DIT [1–6]. DIT in the High-BAT group was 10.3 ± 2.7%, 9.8 ± 2.4%, and 9.3 ± 3.2% after breakfast, lunch, and dinner, respectively, and exhibited no significant differences among the meals. Similarly, DIT in the Low-BAT group exhibited no differences among the meals. When the two groups were compared, DIT after breakfast in the High-BAT group was significantly larger than that in the Low-BAT group (10.3 ± 2.7% vs. 6.9 ± 3.9%; *P* < 0.05). Similar difference in DIT was also found after lunch (*P* = 0.05), but not after dinner (9.3 ± 3.2% vs. 6.7 ± 4.1%; *P* = 0.122). These results suggest that BAT may contribute to DIT after breakfast and lunch more than after dinner.

**Fig. 2:**
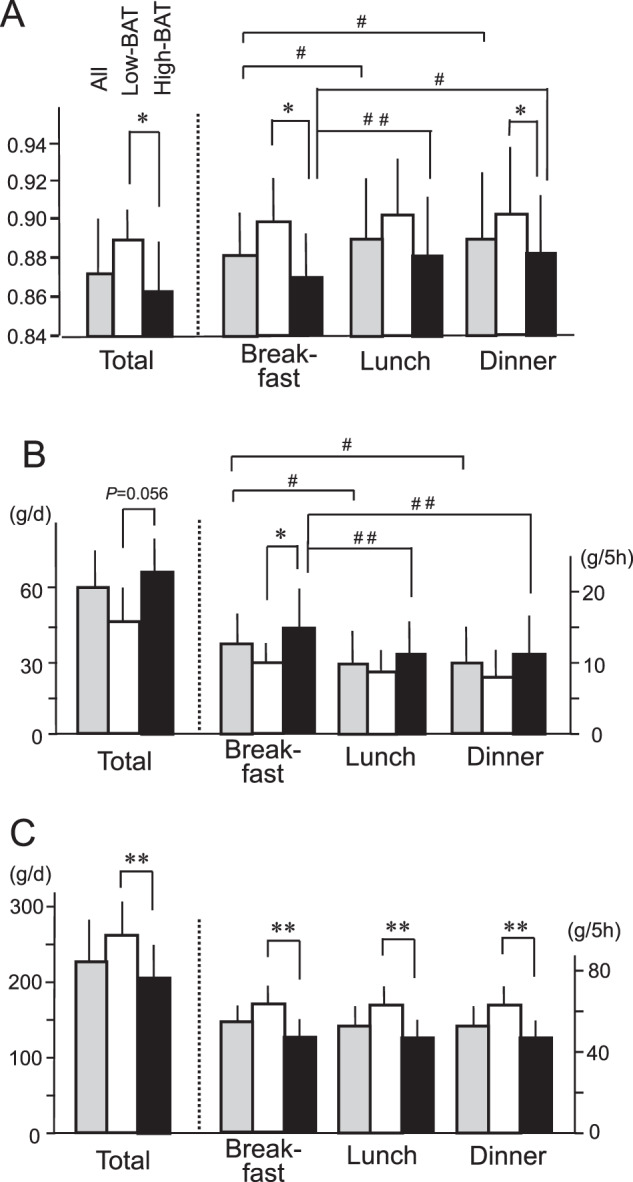
Respiratory quotient and substrate oxidation after breakfast, lunch, and dinner. **A** Respiratory quotient. **B** Fat oxidation. **C** Carbohydrate oxidation. Total is the value per day, and breakfast, lunch, and dinner are the values for 5 h after each meal. Black columns, High-BAT group; white columns, Low-BAT group; gray columns, all. Values are mean ± SD. *P < 0.05 and **P < 0.01 between the groups, ^#^*P* < 0.05 and ^##^*P* < 0.01 between the meals.

The 24 h RQ was significantly lower (*P* < 0.05) in the High-BAT group than in the Low-BAT group (Fig. 2A). In the High-BAT group, the RQ was 0.869 ± 0.029 during a 5 h period after breakfast, and was significantly lower than those after lunch (0.881 ± 0.032, *P* < 0.01) and dinner (0.880 ± 0.032, *P* = 0.05). By contrast, the RQ in the Low-BAT group yielded no significant differences among the meals. Comparison of the two groups indicated that the RQ in the High-BAT group was significantly lower than that in the Low-BAT group after breakfast (*P* < 0.05) and dinner (*P* < 0.05), but not after lunch (*P* = 0.051).

The 24 h fat oxidation calculated from RQ and urinary nitrogen excretion was higher (*P* < 0.05) in the High-BAT group than in the Low-BAT group (Fig. 2B). Notably, fat oxidation in the High-BAT group was higher after breakfast (15.6 ± 5.1 g) than after lunch (11.7 ± 5.5 g, *P* < 0.01) and dinner (11.8 ± 6.0 g, *P* = 0.01). A similar tendency was also found in the Low-BAT group, but the difference did not reach a significant level (*P* = 0.091 for breakfast vs. lunch, and *P* = 0.087 for breakfast vs. dinner). When the two groups were compared, fat oxidation in the High-BAT group was higher than that in the Low-BAT group after breakfast (15.6 ± 5.1 g vs. 10.2 ± 3.0 g, *P* < 0.05), but not after lunch (*P* = 0.143) and dinner (*P* = 0.102). In contrast, carbohydrate oxidation was lower (*P* < 0.05) in the High-BAT group than in the Low-BAT group irrespective of meal timing (Fig. 2C), and exhibited no significant differences among the meals. As such, fat oxidation after breakfast was higher than after lunch and dinner in the High-BAT group, but not in the Low-BAT group, thus suggesting that BAT contributes more to fat oxidation after breakfast and lunch than after dinner.

### STUDY 2: Diurnal changes in cold-induced EE, fat oxidation, and thermogenesis

The results of STUDY 1 of diurnal variations of BAT-associated DIT and fat oxidation suggest that the BAT activity is higher in the morning than in the evening and at night. To confirm this, in STUDY 2, we recruited a new cohort composed of 23 healthy male volunteers, estimated their BAT activities with FDG-PET/CT, and divided them into two Low- and High-BAT activity groups (Table 1). We then measured whole-body EE by indirect calorimetry under a warm condition (27 °C) and 90 min after mild-cold exposure at 19 °C in the morning (0800–1100 h) and evening (1900–2200 h), and calculated CIT as an index of thermogenic activity of BAT.

**Fig. 3:**
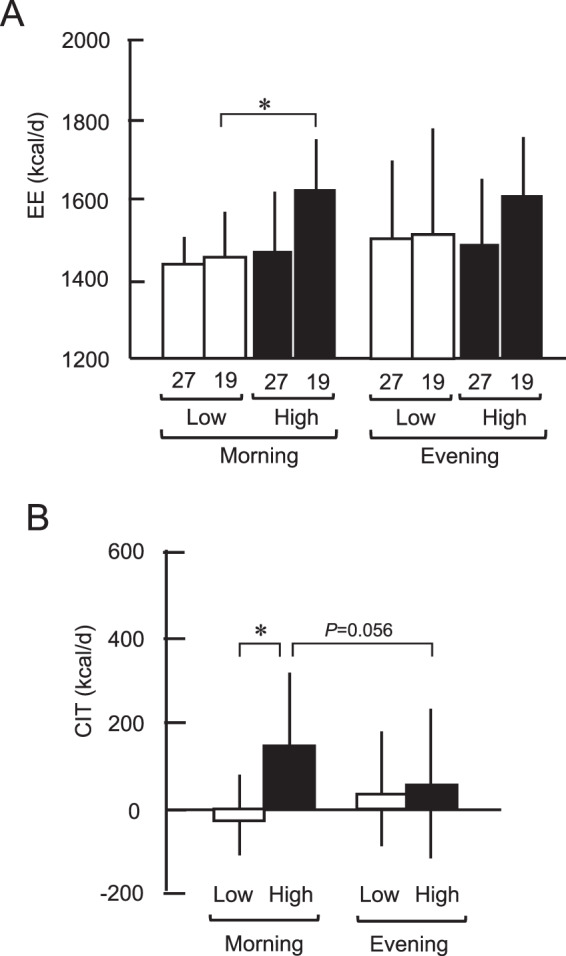
Energy expenditure (EE) at 27 °C (27) and 19 °C (19) in the morning (0800–1100 h) and in the evening (1900–2200 h). **A** Total EE. **B** Cold-induced thermogenesis (CIT). Black columns, High-BAT group; white columns, Low-BAT group. Values are mean ± SD. *P < 0.05 between the groups.

In the morning, whole-body EE at 27 °C was almost equal in the two groups, whereas that at 19 °C slightly increased, being higher in the High-BAT group that in the Low-BAT group (*P* < 0.05) (Fig. 3A). In the evening, however, EE at either 27 °C or 19 °C yielded no significant difference between the two groups. CIT, calculated as the difference in EE at 19 °C and 27 °C, in the High-BAT group was higher than that in the Low-BAT group in the morning (152 ± 167 kcal/d vs. −10 ± 133 kcal/d, *P* < 0.05), but not in the evening (75 ± 154 kcal/d vs. 36 ± 155 kcal/d) (Fig. 3B). Moreover, when compared with the evening CIT, the morning CIT tended to be higher in the High-BAT group (*P* = 0.056), but not in the Low-BAT group (Fig. 3B).

**Fig. 4:**
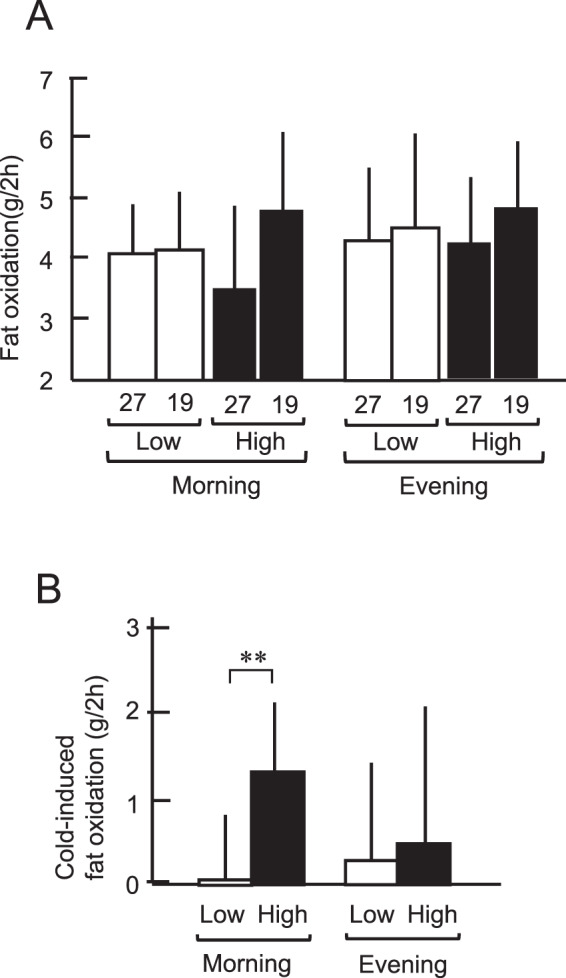
Fat oxidation at 27 °C (27) and 19 °C (19) in the morning (0800h–1100 h) and in the evening (1900h–2200 h). **A** Total fat oxidation. **B** Cold-induced fat oxidation. Black columns, High-BAT group; white columns, Low-BAT group. Values are mean ± SD. **P < 0.01 between the groups.

RQ was almost equal in the two groups and in the morning and evening. Fat oxidation seems to increase slightly at 19 °C (Fig. 4A). The cold-induced fat oxidation, calculated as the difference in fat oxidation at 19 and 27 °C, was higher in the High-BAT group than that in the Low-BAT group only in the morning (1.32 ± 0.78 g vs. 0.02 ± 0.83 g, *P* < 0.01), but not in the evening (0.48 ± 1.60 g vs. 0.31 ± 1.21 g) (Fig. 4B). Thus, cold-induced increase in EE and fat oxidation was higher in the morning than in the evening, and the diurnal difference was dependent on the presence of active BAT. These results strongly support the idea of diurnal variations of BAT with higher activities in the morning.

**Fig. 5:**
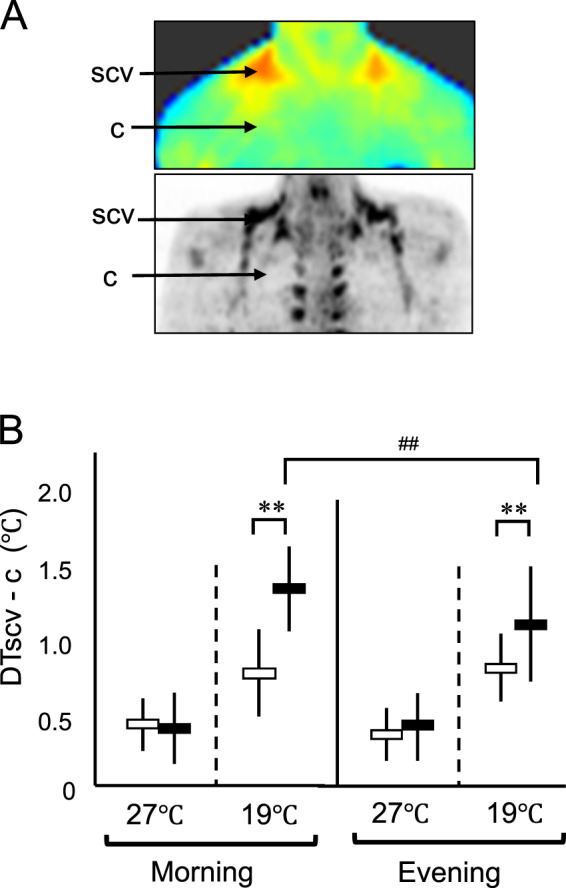
Skin temperature at the supraclavicular (scv) and chest (c) regions at 27 °C and 19 °C in the morning (0800–1100 h) and evening (1900–2200 h). **A** Regions where the measurement of skin temperature was conducted. **B** Difference between Tscv and Tc (DTscv-c) in the morning and evening. Black bars, High-BAT group; white bars, Low-BAT group. Values are the mean ± SD. **P < 0.01 between the groups, ^##^*P* < 0.01 between morning and evening.

In parallel with indirect calorimetry, we also measured skin temperature in the supraclavicular region (Tscv) and in the control chest region (Tc) at 27 °C and following a 90 min cold exposure at 19 °C (Fig. 5A), and calculated the difference between Tscv and Tc (DTscv-c) as another index of thermogenic activity of BAT. As shown in Fig. 5B, the Tscv was 0.35–0.41 °C higher than the Tc at 27 °C. After cold exposure, Tscv dropped in the Low-BAT group, but only slightly in the High-BAT group, whereas Tc dropped considerably and similarly in the two groups. As a result, DTscv-c at 19 °C was larger in the High-BAT group than in the Low-BAT group irrespective of the clock time (*P* < 0.01). In the High-BAT group, the DTscv-c at 19 °C was 1.37 ± 0.29 °C in the morning, which was significantly larger than those in the evening (1.17 ± 0.39 °C, *P* < 0.01). In the Low-BAT group, however, the DTscv-c at 19 °C was comparable in the morning and evening (0.79 ± 0.27 °C and 0.80 ± 0.22 °C). Thus, cold-induced change in DTscv-c was higher in the morning than in the evening, and the diurnal difference was dependent on the presence of active BAT.

## Discussion

We previously measured EE for 24 h with a whole-room indirect calorimeter and found that DIT and fat oxidation are higher in subjects with metabolically active BAT than in those without it, thus suggesting a significant contribution of BAT to DIT in humans [21]. In the present study (STUDY 1), first, we reanalyzed our previous 24 h EE data, focusing on DIT and fat oxidation after breakfast, lunch, and dinner. Although DIT exhibited no significant difference among the meals, DIT after breakfast in the High-BAT group was significantly larger than that in the Low-BAT group. Similar difference in DIT was also found after lunch, but not after dinner. Fat oxidation after breakfast was higher than after lunch and dinner in the High-BAT group, but not in the Low-BAT group. These results suggest that BAT may contribute to DIT and fat oxidation after breakfast and lunch more than after dinner. The diurnal variations of BAT-associated DIT and fat oxidation imply that the BAT activity is higher in the morning than in the evening and at night.

The best protocol for testing the diurnal variation of BAT activity may be repeated FDG-PET/CT scans in different clock times, because FDG-PET/CT is accepted as the standard method for the assessment of BAT. However, the protocol of repeated FDG-PET/CT in a day is strictly limited largely because of radiation exposure, and is not approved by the institutional review boards. We and others have demonstrated that the increment of whole-body EE in response to mild-cold exposure (CIT) is proportional to the BAT activity assessed by FDG-PET/CT [19, 27]. Moreover, it has been reported that the cold-induced change in skin temperature at the supraclavicular region proximal to the underlying BAT depots (Tscv) is positively correlated to the BAT activity assessed by FDG-PET/CT [25, 28, 29]. It is thus feasible to use the cold-induced changes in EE and Tscv as possible surrogates of activated BAT. In the present study (STUDY 2) for a cohort different from STUDY 1, we found cold-induced increases in EE, fat oxidation and Tscv were higher in the morning than in the evening in the High-BAT group, but not in the Low-BAT group. Thus, the diurnal difference was dependent on the presence of active BAT, indicating that the BAT activity is higher in the morning than in the evening.

A circadian rhythm in BAT thermogenesis has well been documented in rodents. That is, thermogenic activities and expression of some key molecules, including thermogenic uncoupling protein 1 (UCP1) in BAT show clear diurnal rhythmic variations that contribute to body temperature and metabolism rhythms of glucose and lipids [30, 31]. In addition to UCP1, various clock-related molecules, such as Bmal1, Per(s), and Cry(s), show clear circadian rhythms, and the nuclear receptor Rev-erbα plays a critical role in the control of the circadian thermogenic rhythm of BAT [32]. In humans, Lee et al. [33] reported circadian rhythmic changes in glucose uptake and in mRNA expressions of UCP1, Glut 4, and Rev-erbα in adipocytes, and in adipose explants isolated from the neck regions and cultured in vitro, suggesting an autonomous cell thermogenic rhythm pertaining to human BAT. Our results are well consistent with their in vitro observations, and demonstrated diurnal changes of human BAT in vivo.

As noted above, an intrinsic cell-autonomous circadian mechanism is involved in the rhythmic changes of BAT. However, it is also possible that the mechanism can be modified by various neuroendocrine factors. The sympathetic nervous system (SNS) is the most likely regulatory mechanism because the SNS is critical to activate BAT thermogenesis in vivo. In fact, Orozco-Solis et al. demonstrated in mice that the circadian clock in the ventromedial hypothalamus (VMH) controls circadian EE through the rhythmic activation of BAT via the SNS [34]. This is consistent with the view that VMH is intimately associated with sympathetic facilitation in peripheral tissues including BAT [35, 36]. Another possible factor may be glucocorticoids [37]. Plasma cortisol levels are highest in the early morning and decrease in the evening. Considering the acute stimulatory effect of glucocorticoids on BAT in humans [38], it may be conceivable that the cortisol surge in the morning could potentially increase BAT activity. It is also possible that the responsiveness of BAT to catecholamines and glucocorticoids may diurnally change. Additional studies are required to clarify the physiological mechanisms that underlie the human BAT rhythm.

The major fuel substrate of BAT thermogenesis is fatty acids derived not only from intracellular triglyceride but also from blood lipoproteins and nonesterified fatty acids (NEFA), implying BAT as a metabolic sink and regulator of plasma lipids [39, 40]. Din et al. [22] demonstrated in humans that postprandial NEFA uptake into BAT was minimal compared with that after cold exposure, but it was still proportional to BAT thermogenesis. Moreover, van den Berg et al. [41]. reported that a diurnal rhythm in BAT causes rapid clearance and combustion of plasma lipids at the start of dark period in mice. In humans, postprandial NEFA levels were lower in the morning than in the evening. These results seem consistent with our present finding of the diurnal variation of BAT activity with higher fatty acid oxidation in the morning.

The present study demonstrated a diurnal variation in fat oxidation, but not in carbohydrate oxidation, in the High-BAT group, suggesting that BAT utilizes fatty acids more preferentially in the morning than in the evening. Recently, we [42] reported a role of Kruppel-like factor 15 (KLF15), a transcription factor highly expressed in adipose tissue, in the regulation of fuel switching between glucose and fatty acids in response to changes in energy status in BAT. It is also known that KLF15 expression exhibits a clear circadian rhythm in several peripheral organs and coordinates diurnal variations of plasma levels and catabolism of amino acids including branched-chain amino acids (BCAA) [43]. In this context, interesting is that BAT actively utilizes BCAA for thermogenesis and promotes systemic BCAA clearance [44]. To date, it is unknown whether BCAA metabolism in BAT exhibits diurnal variations, but it is conceivable that KLF may be a critical regulator of the diurnal variation of substrate utilization in BAT.

In conclusion, we demonstrated in healthy human subjects that NST and fat oxidation after either cold exposure or meal intakes are high in the morning compared with the evening in subjects with High-BAT activity, but not in those with Low-BAT activity. This suggests that the activities of human BAT exhibit diurnal changes, being high in the morning compared in the evening. Considering the beneficial effects of BAT on body fatness, insulin sensitivity, macronutrient metabolism and cardiovascular functions [45, 46], the present results may explain, at least in part, the apparent association of meal timing with obesity and related metabolic disorders [7–11]. For example, habits of breakfast skipping and overeating at night may result in relatively less BAT activation and decreased fat oxidation, leading increased body fat accumulation. Recently, time-restricted feeding (TRF), an eating pattern that limits food intake to a specific period of 4–12 h without altering nutrient quality or quantity, is emerging as a therapeutic strategy against obesity and related metabolic disorders [47]. The metabolic effects of TRF are thought to be based on the realignment of feeding and the circadian clock, which improves nutrient utilization and EE. Indeed, TRF results in increased and rhythmic expression of UCP1 in parallel with increased EE [48, 49]. Moreover, in nocturnal mice the effects of TRF are greater when feeding time is restricted during the active (dark) phase than during the inactive (light) phase [50, 51]. All these results, together with those of our present study, suggest that in diurnal humans TRF in the morning and/or during the daytime is more effective for BAT activation and beneficial than in the evening and/or during the night time.
